# Concentration-dependent alpha_1_-Adrenoceptor Antagonism and Inhibition of Neurogenic Smooth Muscle Contraction by Mirabegron in the Human Prostate

**DOI:** 10.3389/fphar.2021.666047

**Published:** 2021-06-24

**Authors:** Ru Huang, Yuhan Liu, Anna Ciotkowska, Alexander Tamalunas, Raphaela Waidelich, Frank Strittmatter, Christian G. Stief, Martin Hennenberg

**Affiliations:** Department of Urology, University Hospital, LMU Munich, Munich, Germany

**Keywords:** mirabegron, lower urinary tract symptoms (LUTS), benign prostatic hyperplasia (BPH), obstructive symptoms, bladder outlet obstruction (BOO), prostate smooth muscle contraction, α1-adrenoceptor

## Abstract

**Introduction:** Mirabegron is available for treatment of storage symptoms in overactive bladder, which may be improved by β_3_-adrenoceptor-induced bladder smooth muscle relaxation. In addition to storage symptoms, lower urinary tract symptoms in men include obstructive symptoms attributed to benign prostatic hyperplasia, caused by increased prostate smooth muscle tone and prostate enlargement. In contrast to the bladder and storage symptoms, effects of mirabegron on prostate smooth muscle contraction and obstructive symptoms are poorly understood. Evidence from non-human smooth muscle suggested antagonism of α_1_-adrenoceptors as an important off-target effect of mirabegron. As α_1_-adrenergic contraction is crucial in pathophysiology and medical treatment of obstructive symptoms, we here examined effects of mirabegron on contractions of human prostate tissues and on proliferation of prostate stromal cells.

**Methods:** Contractions were induced in an organ bath. Effects of mirabegron on proliferation, viability, and cAMP levels in cultured stromal cells were examined by EdU assays, CCK-8 assays and enzyme-linked immunosorbent assay.

**Results:** Mirabegron in concentrations of 5 and 10 μM, but not 1 µM inhibited electric field stimulation-induced contractions of human prostate tissues. Mirabegron in concentrations of 5 and 10 µM shifted concentration response curves for noradrenaline-, methoxamine- and phenylephrine-induced contractions to the right, including recovery of contractions at high concentrations of α_1_-adrenergic agonists, increased EC_50_ values, but unchanged E_max_ values. Rightshifts of noradrenaline concentration response curves and inhibition of EFS-induced contractions were resistant to L-748,337, l-NAME, and BPIPP. 1 µM mirabegron was without effect on α_1_-adrenergic contractions. Endothelin-1- and U46619-induced contractions were not affected or only inhibited to neglectable extent. Effects of mirabegron (0.5–10 µM) on proliferation and viability of stromal cells were neglectable or small, reaching maximum decreases of 8% in proliferation assays and 17% in viability assays. Mirabegron did not induce detectable increases of cAMP levels in cultured stromal cells.

**Conclusion:** Mirabegron inhibits neurogenic and α_1_-adrenergic human prostate smooth muscle contractions. This inhibition may be based on antagonism of α_1_-adrenoceptors by mirabegron, and does not include activation of β_3_-adrenoceptors and requires concentrations ranging 50-100fold higher than plasma concentrations reported from normal dosing. Non-adrenergic contractions and proliferation of prostate stromal cells are not inhibited by mirabegron.

## Introduction

Mirabegron is a β_3_-adrenoceptor agonist, and has been approved for medical treatment of storage symptoms due to overactive bladder (OAB) (Oelke et al., 2013; Chapple et al., 2014; Nambiar et al., 2018). Storage symptoms are caused by involuntary detrusor contractions (Andersson and Arner, 2004). In view that mirabegron inhibits bladder smooth muscle contractions *in vitro*, symptom improvements by mirabegron may result from direct β_3_-adrenoceptor-induced smooth muscle relaxations in the bladder wall (Andersson and Arner, 2004; Takasu et al., 2007; Svalo et al., 2013). In men, lower urinary tract symptoms (LUTS) include obstructive symptoms, which are commonly attributed to benign prostatic hyperplasia (BPH) and may occur alone or together with storage symptoms (Oelke et al., 2013). Increased prostate smooth muscle tone in BPH holds a central role in pathophysiology and medical treatment of obstructive symptoms (Oelke et al., 2013; Hennenberg et al., 2014). Thus, impaired voiding and impaired bladder emptying in BPH may be caused by urethral obstruction due to increased prostate smooth muscle tone (Oelke et al., 2013; Hennenberg et al., 2014). Consequently, drugs for medical treatment of LUTS suggestive of BPH include α_1_-adrenoceptor antagonists (α_1_-blockers) as the first-line option and the phosphodiesterase five inhibitor tadalafil, which both inhibit prostate smooth muscle contraction and are applied for rapid symptom improvement (Michel and Vrydag, 2006; Oelke et al., 2013). Apart from intraprostatic smooth muscle tone, prostate growth may contribute to urethral compression as well in BPH, so that 5α-reductase inhibitors are used to reduce prostate growth and to prevent disease progression and complications (Oelke et al., 2013; Hennenberg et al., 2014).

Following its approval for treatment of storage symptoms, possible actions of mirabegron on BPH-related symptoms have been considered. However, available findings from clinical and preclinical studies are conflicting. Clinical trials mostly demonstrated that mirabegron does not improve obstructive symptoms in BPH (Nitti et al., 2013; Liao and Kuo, 2018), but are contrasted by few experimental studies suggesting that mirabegron induces smooth muscle relaxation of prostate tissues *in vitro* (Calmasini et al., 2015; Alexandre et al., 2016). Thus, the actions of mirabegron on the prostate are poorly understood, although the drug is clinically used in patients with LUTS. Previous studies addressing effects of mirabegron on prostate smooth muscle contraction were mostly based on rodent tissues and included only one series using human tissues and no data regarding neurogenic or non-adrenergic contractions (Calmasini et al., 2015; Alexandre et al., 2016). Non-adrenergic smooth muscle contractions in the prostate can be induced by endothelin-1 or thromboxane A_2_, and have been supposed to contribute to prostate smooth muscle tone in parallel to α_1_-adrenoceptors, or even to maintain urethreal obstruction during treatment with α_1_-blockers (Hennenberg et al., 2014). β_3_-Adrenergic smooth muscle relaxation may principally occur by formation of the second messenger cAMP, but may involve cAMP-independent mechanisms and nitric oxide/cGMP-mediated relaxation as well (Flacco et al., 2013; Bussey et al., 2018; Maki et al., 2019; Okeke et al., 2019). Prostate growth, in turn, is largely androgen-dependent and may be involve stromal and epithelial hyperplasia (Strand et al., 2017). In addition, α_1_-adrenoceptor-mediated proliferation of prostate cells has been repeatedly suggested, although α_1_-blockers do not reduce prostate volume in patients with BPH (Oelke et al., 2013). Considering that β_3_-adrenergic proliferation has been reported from non-prostatic smooth muscle cells (Hadi et al., 2013), β_3_-adrenergic or mirabegron-induced proliferation of prostate smooth muscle cells appears generally possible as well, but has not yet been addressed.

Although the affinity of mirabegron is highest for β_3_-adrenoceptors, off-target effects became obvious following its introduction for treatment of storage symptoms (Dehvari et al., 2018; Igawa et al., 2019; Michel, 2020). Specifically, antagonism of α_1_-adrenoceptors by mirabegron has been proposed, which was mostly based on evidence from non-human tissues, including the urethra, prostate, vas deferens, and aorta (Alexandre et al., 2016; Michel, 2020). Assuming that this antagonism also occurs in the prostate and acknowledging that α_1_-adrenoceptors are an important target for medical treatment in BPH, it can presently hardly explained why the improvements of LUTS by mirabegron are apparently restricted to storage symptoms and do not include obstructive symptoms. Regarding that mirabegron is available for clinically used for LUTS treatment in at least several countries and regions, while its actions are incompletely understood and off-target effects are obvious, improved understanding of its actions in the lower urinary tract appears appropriate. Here, we addressed the effects of mirabegron on human prostate smooth muscle contraction, induced by neurogenic stimulation, adrenergic agonists and non-adrenergic mediators, and using different mirabegron concentrations. In parallel, we examined effects of mirabegron on proliferation of prostate stromal cells, as adrenoceptors were repeatedly suspected to regulate cell cycle and growth.

## Materials and Methods

### Human Prostate Tissues

Human prostate tissues were obtained from patients who underwent radical prostatectomy for prostate cancer (*n* = 109). Patients with previous transurethral resection of the prostate were excluded. This study was carried out in accordance with the Declaration of Helsinki of the World Medical Association and has been approved by the ethics committee of the Ludwig-Maximilians University, Munich, Germany. Informed consent was obtained from all patients. All samples and data were collected and analyzed anonymously. Accordingly, not patients’ data were analyzed or related with sampled tissues. Following removal of prostates from patients, macroscopic pathologic examination and sampling were performed within approximately 30 min. Organ bath studies were started within 1 h following sampling, i. e. approximately 1.5 h following surgical removal of the organs. For transport and storage, organs and tissues were stored in Custodiol^®^ solution (Köhler, Bensheim, Germany). For macroscopic examination and sampling of prostate tissues, the prostate was opened by a single longitudinal cut reaching from the capsule to the urethra. Subsequently, both intersections were checked macroscopically for any obvious tumor infiltration. Tissues were taken solely from the transitional, periurethral zone, considering the fact that most prostate cancers arise in the peripheral zone (Pradidarcheep et al., 2011; Shaikhibrahim et al., 2012). In fact, tumor infiltration in the periurethral zone (where sampling was performed) was very rare (found in less than 1% of prostates). Prostates showing tumors in the periurethral zone upon macroscopic inspection were not subjected to sampling and were not included in this study. BPH is present in ca. 80% of patients with prostate cancer (Alcaraz et al., 2009; Orsted and Bojesen, 2013). The age of patients undergoing radical prostatectomy at our department averages out at 66 ± 7 years (mean ± standard deviation, *n* = 4,003 patients) (Grabbert et al., 2018), where the prevalence of histological BPH may range between 60 and 70% (Lepor, 2004). Typically, most tissues previously sampled under the same conditions in our studies showed a prostate-characteristic architecture with composition of glands and smooth muscle-rich stroma (Wang et al., 2015; Wang et al., 2016), while tissue containing only stroma without glands is usually limited to the anterior parts of the human prostate (Chakrabarty et al., 2019).

### Organ Bath

Prostate strips (6 × 3 × 3 mm) were mounted in 10 ml aerated (95% O_2_ and 5% CO_2_) tissue baths (Danish Myotechnology, Aahus, Denmark) with four chambers, containing Krebs-Henseleit solution (37 C, pH 7.4). Preparations were stretched to 4.9 mN and left to equilibrate for 45 min. In the initial phase of the equilibration period, spontaneous decreases in tone are usually observed. Therefore, tension was adjusted three times during the equilibration period, until a stable resting tone of 4.9 mN was attained. After the equilibration period, maximum contraction induced by 80 mM KCl was assessed. Subsequently, chambers were washed three times with Krebs-Henseleit solution for a total of 20 min, followed by addition of mirabegron (1 μM, 5 µM or 10 µM) or equivalent amounts of solvent for controls. Cumulative concentration response curves for contractile agonists, or frequency response curves for electric field stimulation (EFS) were constructed 30 min after addition of mirabegron or solvent. Concentration ranges for contractile agonists included 0.1–100 µM for all three α_1_-adrenergic agonists (noradrenaline, methoxamine, phenylephrine), 0.01–30 µM for U46619, and 0.01–3 µM for endothelin-1. Application of EFS simulates action potentials, resulting in contractions of human prostate tissues by the release of endogenous neurotransmitters, including noradrenaline. Accordingly, tetrodotoxin inhibited EFS-induced contractions under our and similar conditions, as previously reported (Yu et al., 1994; Angulo et al., 2012; Li et al., 2020; Spek et al., 2021). For EFS, tissue strips were placed between two parallel platinum electrodes connected to a CS4 stimulator (Danish Myotechnology, Denmark). Square pulses with durations of 1 ms were applied with a voltage of 20 V, for a train duration of 10 s. EFS-induced contractile responses were studied at frequencies of 2, 4, 8, 16, and 32 Hz, with train intervals of 30 s between stimulations.

In further experiments, EFS- and noradrenaline-induced contractions (frequency and concentration response curves) were induced after addition of mirabegron (10 µM) or solvent as described above, but in the presence of L-748,337 (1 µM), l-NAME (200 µM) or BPIPP (30 µM) in all chambers of one experiment. Thus, one of these compounds was added to all four tissues of the same experiment, following washout of KCl and 20 min before addition of mirabegron or solvent. In another series of experiments, tissues were contracted with 80 mM KCl as described above. However, instead of washing out KCl after the maximum contraction was reached, cumulative concentrations of mirabegron were added after a stable precontraction was attained. Typically, the stable tension has been reached within approximately 10 min after addition of KCl, either after a more or less pronounced maximum peak contraction, or following a continous increase to the plateau without peak contraction. The first two concentrations (1 μM, 3 µM) were left for 5 min, until the last concentration was applied (10 µM) and left for further 10 min, i. e. until the end of the experiment.

Each independent experiment was performed using tissue from one prostate, which was examined with mirabegron and as control group (DMSO as solvent for mirabegron). Only one concentration response or frequence response curve was recorded with each tissue. Wherever possible, double determinations were performed. For double determinations, two from the four organ bath channels filled with tissue from the same prostate were examined with mirabegron, and the two others were used as controls (DMSO). From a total of 104 organ bath experiments including frequency and concentration response curves with two groups (mirabegron and control), double determinations in both groups were possible in 86 experiments. In the remaining experiments, the amount of sampled tissues did not allow filling of two channels for both groups, so that single determinations were peformed in one group, or rarely in both groups. However, each experiment contained at least one sample for both groups, resulting in paired samples. Allocations to the control and drug group were changed for each experiment. For each but one series (as indicated), five of such independent experiments were performed. In experiments addressing mirabegron effects on precontracted tissues, double determination was possible in four out of five experiments.

For calculation of EFS- and agonist-induced contractions, tensions were expressed as percentage of maximum 80 mM KCl-induced contractions, as this may correct different stromal/epithelial ratios and different smooth muscle content, resulting from varying phenotypes and degrees of BPH, or from any other individual variation and heterogeneity between prostate samples and patients. In fact, normalization to KCl allows to examine possible alterations of receptor responsiveness, whereas correlations between agonist-induced force and ring weight, length, or cross-sectional area may be weak or lacking in organ bath experiments, at least in some tissue types (Erdogan et al., 2020). In addition to construction of concentration response curves, EC_50_ values for contractile agonists and E_max_ values were calculated by curve fitting. Curve fitting was performed for each single experiment, using GraphPrism 6 (Statcon, Witzenhausen, Germany), and values were analyzed as described below. In experiments addressing effects of mirabegron on KCl-precontracted tissues, tensions after mirabegron are expressed as percentage of the stable, KCl-induced precontration (text) and as percentage decrease from precontraction (figure).

### Cell Culture

WPMY-1 cells are a SV40 large-T antigen-immortalized cell line, obtained from the stroma of a human prostate without prostate cancer (Webber et al., 1999). According to the typical composition of the prostate stroma, where smooth muscle cells are the predominant cell type, WPMY-1 cells show characteristics of myofibroblasts and prostate smooth muscle cells, including expression of vimentin, *a*-smooth muscle actin (α-SMA), calponin and α_1A_-adrenoceptors, but lacking expression of cytokeratins and tyrosine hydroxylase (Webber et al., 1999; Wang et al., 2015; Wang et al., 2016). WPMY-1 cells were purchased from the American Type Culture Collection (ATCC; Manassas, VA, United States of America), and cultured in RPMI 1640 (Gibco, Carlsbad, CA, United States) supplemented with 10% fetal calf serum (FCS) and 1% penicillin/streptomycin at 37 C with 5% CO_2_. Before addition of mirabegron or DMSO (solvent for mirabegron) to cells, the medium was changed to a FCS-free medium.

### Proliferation Assay

Proliferation rate of cells was assessed using the 5-ethynyl-2’-deoxyuridine- (EdU-)based EdU-Click 555 proliferation assay kit (Baseclick, Tutzing, Germany), which was applied according to the manufacturer’s instructions. In this assay, incorporation of EdU into DNA of proliferating cells is assessed by detection with fluorescing 5-carboxytetramethylrhodamine (5-TAMRA). 30,000 cells were placed in each well of a 16-well chambered coverslip (Thermo Scientific, Waltham, MA, United States), and cultured for 24 h. Subsequently, the medium was replaced by 10 mM EdU solution in FCS-free smooth muscle cell medium containing mirabegron or DMSO. 24 h later, cells were fixed with ROTI^®^ Histofix 4% solution (Roth, Karlsruhe, Germany). Counterstaining of all nuclei was performed with DAPI. Finally, analysis was performed by fluorescence microscopy (excitation: 546 nm; emission: 479 nm) using a laser scanning microscope (Leica SP2, Wetzlar, Germany). Stainings were quantified using “ImageJ” (National Institutes of Health, Bethesda, Maryland, United States).

### Viability Assay

Viability was assessed using the Cell Counting Kit-8 (CCK-8) (Sigma-Aldrich, Munich, Germany). Cells were seeded in 96-well plates (5,000 cells/well), and cultured for 24 h. Subsequently, mirabegron in indicated concentrations or DMSO in corresponding amounts were added, and cells were cultured for further 48 h until assessment. Finally, 10 μl of [2-(2-methoxy-4-nitrophenyl)- 3-(4-nitrophenyl)-5-(2,4-disulfophenyl)-2H-tetrazolium monosodium salt (WST-8) from the kit were added, and absorbance in each well was measured at 450 nm after incubation for 2 h at 37 C.

### cAMP ELISA

For each sample, 10^6^ cells were seeded to cell culture flasks and cultured for two days in RPMI containing 10% FCS (12 ml). Three days later, the medium was changed to medium without FCS (12 ml) after washing with phosphate-buffered saline (PBS), and mirabegron (in final concentrations of 1 µM or 10 µM) or DMSO in corresponding amounts were added. After 30 min, cells were lyzed for 10 min with 0.1 M HCl, centrifuged, and supernatants were subjected to cAMP determination using an enzyme-linked immunosorbent assay (ELISA) (ADI-900-066, Enzo Life Sciences, Farmingdale, NY, United States), according to the manufacturer’s instructions and using acetylation. All samples were measured twice, i. e. in double determinations.

### Materials, Drugs, and Nomenclature

Mirabegron (2-Amino-N-[4-[2-[[(2 R)-2-hydroxy-2-phenylethyl]amino]ethyl]phenyl]-4-thiazoleacetamide) is an adrenoceptor ligand, which is available as a β_3_-adrenoceptor agonist for medical treatment of storage symptoms due to overactive bladder. In fact, its affinity is highest for β_3_-adrenoceptors, although its selectivity has been questioned, as explained in the introduction and discussion. Stock solutions (10 mM) were prepared with DMSO, and aliquots were stored at -20°C until use. L-648,377 (N-[[3-[(2S)-2-Hydroxy-3-[[2-[4-[(phenylsulfonyl)amino]phenyl]ethyl]amino]propoxy]phenyl]methyl]-acetamide) is a β-adrenoceptor antagonist, with K_i_ values of 4, 204, and 390 nM for β_3_-, β_2_-and β_1_-adrenoceptors, respectively (Candelore et al., 1999). Stock solutions (10 mM) were prepared with DMSO, and aliquots were stored at -20 C until use. Nω-nitro-L-arginine methyl ester (l-NAME) acts as an ubiquitous inhibitor of nitric oxide synthases (NOS) following hydrolysis by intracellular esterases and was applied using a final concentration of 200 μM, which inhibits all three NOS isoforms. Aqueous stock solutions (10 mM) were freshly prepared before each experiment. BPIPP 5-(3-bromophenyl)-5,11-dihydro-1,3-dimethyl-1H-indeno [2′,1':5,6]pyrido [2,3 days]pyrimidine-2,4,6(3H)-trione] is a non-competitive inhibitor of guanylyl and adenylyl cyclases, which inhibits cGMP synthesis induced by nitric oxide or natriuretic peptides, and forskolin- and isoproterenol-induced cAMP synthesis (Kots et al., 2008). Stock solutions (2 mM) were freshly prepared with DMSO before each experiment. Phenylephrine (R)-3-[-1-hydroxy-2-(methylamino)ethyl]phenol and methoxamine (α-(1-Aminoethyl)-2,5-dimethoxybenzyl alcohol) are α_1_-selective adrenoceptor agonists. Aqueous stock solutions (10 mM) of noradrenaline, phenylephrine, and methoxamine were freshly prepared before each experiment. Aqueous stock solutions of endothelin-1 (0.4 mM) were stored at −20°C as small aliquots, so that repeating freezing and thawing cycles were avoided. U46619 ((Z)-7-[(1S,4R,5R, 6S)-5-[(E, 3S)-3-hydroxyoct-1-enyl]-3-oxabicyclo [2.2.1]heptan-6-yl]hept-5-enoic acid) is an agonist of the TXA_2_ receptor and was dissolved in ethanol. As thromboxane A_2_ is highly unstable, U46619 is commonly used as a thromboxane A_2_ receptor agonist. Stock solutions (10 mM) were stored at −80°C until use. Miragebron was obtained from Tocris (Bristol, United Kingdom). U46619 and endothelin-1 were obtained from Enzo Life Sciences (Lörrach, Germany). L-NAME, Noradrenaline, phenylephrine, and methoxamine were obtained from Sigma-Aldrich (Munich, Germany).

### Data and Statistical Analyses

Data in concentration and frequence response curves are presented as means ± standard deviation (SD) with the indicated number (n) of independent experiments. One-way analysis of variance (ANOVA) was used for comparison of whole concentration/frequence response curves, and two-way ANOVA was used for comparison of contractions at single concentrations. Data of EC_50_ and E_max_ values and from cell culture experiments are presented in scatter plots, including all single values. For comparison of paired groups in datasets containing E_max_ and EC_50_ values, a paired Student’s t-test was applied. Tensions following addition of mirabegron on precontracted tissues were compared to tensions before addition of mirabegron by a Dunn’s test, as non-parametric comparison has been recommended to normalized data including a control group without variance (Curtis et al., 2018). Apart from cAMP measurements, multiple groups in cell culture experiments were compared to one control by a Dunnett’s test, which allows comparison of a number of treatments with a single control. Values from cAMP measurements were compared by paired Student’s t-test. All tests were performed using the SPSS^®^ version 20 (IBM SPSS Statistics, IBM Corporation, Armonk, New York, United States), with the exception of Dunn’s test, which was performed using GraphPrism6. *p* values < 0.05 were considered significant. In parallel to concentration and frequence response curves and scatter plots in figures, effect sizes are reported in the text and tables, according to recent recommendations (Michel et al., 2020). Mean differences (MD) with 95% confidence intervals (CI) were calculated for tensions at each frequence and agonist concentration and are reported in tables. MDs with 95% CIs for EC_50_ values were calculated using SPSS^®^ version 20. Calculation of effect sizes in EFS-induced contractions and in assays for proliferation and viability were based normalization of values in inhibitor groups to corresponding value in the control group in the same experiment, and formation of means and 95% CIs from these values.

The present study and analyses have exploratory character, but are not designed to test a pre-specified, or statistical null hypothesis (Michel et al., 2020). In fact, important features of hypothesis-testing studies are lacking, including definition of a tested hypothesis, blinding or a preset study plan based on biometric calculation of groups sizes, as defined by recent guidelines (Michel et al., 2020). According to these guidelines (Michel et al., 2020), *p* values were used sparingly, so that reporting of *p* values is limited to figures but no *p* values are repeated in the text, and *p* values of 0.05 or higher are not indicated. Consequently, data sets without *p* values do not show significant differences. According to the exploratory study design, *p* values reported here should be considered as descriptive and not as hypothesis-testing (Michel et al., 2020).

The minimum number of experiments and group sizes in organ bath experiments was pre-planned as *n* = 5/group, as a calculation of descriptive *p* values was intended. Thus, data were extracted and analyzed, after five experiments of a series were performed. Originally, we intended to decide whether a series is continued or not following this interim analysis of five independent experiments. Thus, increasing the number of experiments in a series was scheduled, if these initial results did not reveal *p* values < 0.05, but suggested that an effect could be expected. Discontinuation was intended, if no effect was seen in concentration response curves, or if descriptive *p* values < 0.05 were obtained in concentration/frequence response curves (at single frequencies/agonist concentrations, and/or between whole groups). This procedure was possible, as our study was explorative but not designed to test a pre-specified statistical null-hypothesis (Michel et al., 2020). In fact, flexible group sizes have been recommended by guidelines for experimental design and analysis in experimental pharmacology, if data are characterized by large variations, what applies here (Curtis et al., 2015; Curtis et al., 2018). In fact, all except one series of organ bath experiments revealed conclusive findings after five independent experiment. Consequently, all but one groups included in the statistical analyses were based on five independent experiments and included tissues from five patients in each group. The exception is a series containing nine indepdendent experiments, as four further experiments were performed after the results of five initial experiments were of limited conclusiveness. Any comparison (e. g. by statistical tests) was confined to paired samples (i. e. tissues from the same prostate), precluding any comparison between different series. According to the paired design (allocation of samples from each tissue to the control and inhibitor groups), groups being compared with each other by statistical tests showed identical group sizes. Cell culture experiments addressing efffects of mirabegron on cAMP formation included four (instead of five) independent experiments for technical reasons. Thus, the kit size allowed measurement of samples from four, but not five experiments. As the results from four initial experiments did not provide a basis to expect any significant effects of mirabegron, the series was discontinued after these four experiments and not subjected to statistical analysis.

No data or experiments were excluded from analyses. An exception is, to some extent, that values for contraction levels at high agonist concentrations were omitted for curve fitting in three experiments, where maximum contractions in control groups were already attained at moderate concentrations. Thus, curve fitting requires clearly sigmoidal character of concentration response curves. Otherwise, any calculated EC_50_ or E_max_ value will be marked as ambigous (or impossible to obtain). Consequently, only the parts showing sigmoidal character were considered in these three experiments, what was the case in control groups of three experiments with noradrenaline (controls for 10 µM mirabegron without futher interventions, where only ranges from 0.1 to 3 μM and 0.1–30 µM noradrenaline were used for curve fitting in two experiments; control for 10 µM mirabegron with BPIPP, where only the range from 0.1 to 10 µM noradrenaline was used for curve fitting in one experiment), and one experiment with U46619 (only 0.01–3 µM U46619 was used for curve fitting in one experiment). However, for diagrams showing concentration response curves, all values were used.

## Results

### Effects of Mirabegron on Electric Field Stimulation-Induced Contractions

**FIGURE 1:**
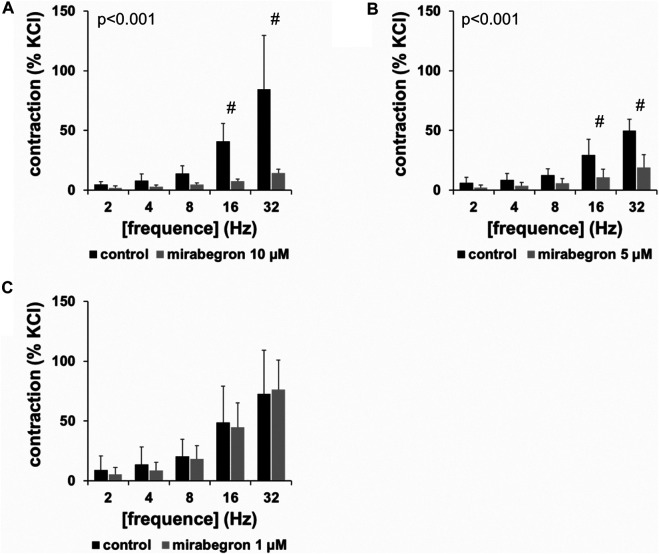
Effects of mirabegron on EFS-induced contractions of human prostate tissues. Contractions of prostate tissues from the periurethral zone were induced by EFS in an organ bath, 30 min after addition of mirabegron in concentrations of 10 µM **(A)**, 5 µM **(B)** or 1 µM **(C)**, or an equivalent amount of DMSO (controls), which was used as solvent for mirabegron. Shown are data from *n* = 5 patients in each diagram, which are means ± SD in frequency response curves (#*p* < 0.05 for control vs. mirabegron by two-way ANOVA, and *p* values for whole groups in inserts from 1-way ANOVA).

EFS induced frequence-dependent contractions of human prostate tissues, which were inhibited by 10 and 5 µM mirabegron, but remained unchanged by 1 µM mirabegron (Figure 1). Inhibitions were clearest at frequencies of 16 and 32 Hz (Figure 1) (Table 1). Contractions were inhibited by 78% [66 to 91] at 16 Hz and by 79% [68 to 90] at 32 Hz by 10 µM mirabegron (Figure 1A), and by 57% [30 to 84] at 16 Hz and by 59% [31 to 86] at 32 Hz by 5 µM mirabegron (Figure 1B).

**TABLE 1:** Mean differences (MD) for EFS-induced contractions of human prostate tissues after application of mirabegron (1, 5, 10 µM) or DMSO for controls, shown for each applied frequence and with 95% confidence intervals (CI) (in square brackets, low to high) (% of KCl-induced contractions). Differences in contraction at given frequencies were calculated for each single experiment (i. e., between mirabegron and control group, for corresponding, paired samples from the same prostate in each single experiment), and are expressed as MD with 95% CI.

Mirabegron (µM)	EFS, frequence				
	2 Hz	4 Hz	8 Hz	16 Hz	32 Hz
**1**	−3.8 [−13.2 to 5.5]	−5.1 [−19.0 to 8.8]	−1.9 [−11.2 to 7.4]	−4.1 [−23.6 to 15.5]	3.6 [−22.1 to 29.2]
**5**	−3.8 [−10.6 to 3.1]	−4.7 [−13.1 to 3.6]	−6.9 [−14.5 to 0.6]	−18.6 [−39.4 to 2.1]	−30.5 [−52.1 to −9.0]
**10**	-2.7 [-5.1 to -0.2]	−5.1 [−11.7 to 1.4]	−9.5 [−16.6 to -2.3]	−33.3 [−53.3 to −13.4]	−70.3 [−128 to −12.7]

Data are based on experiments on series with *n* = 5 prostates for each mirabegron concentration and corresponding controls, i. e. 15 prostates in total.

### Effects of Mirabegron on α_1_-Adrenergic Contractions

**FIGURE 2:**
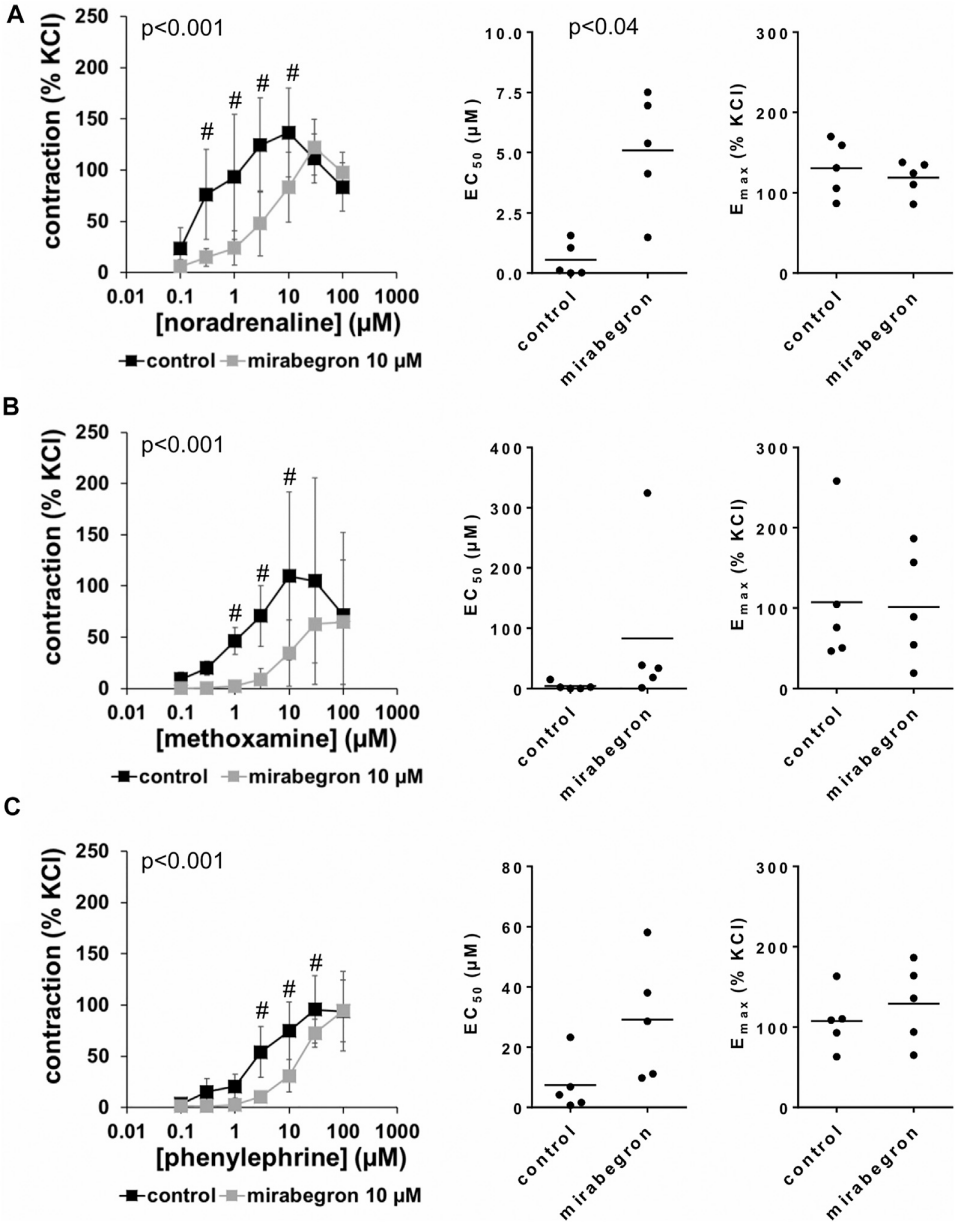
Effects of 10 µM mirabegron on α_1_-adrenergic contractions of human prostate tissues. Contractions of prostate tissues from the periurethral zone were induced by cumulative concentrations of noradrenaline **(A)**, methoxamine **(B)** or phenylephrine **(C)** in an organ bath, 30 min after addition of mirabegron or an equivalent amount of DMSO (controls), which was used as solvent for mirabegron. Shown are data from *n* = 5 patients in each diagram, which are means ± SD in frequency response curves (#*p* < 0.05 for control vs. mirabegron by two-way ANOVA, and *p* values for whole groups in inserts from 1-way ANOVA), and E_max_ and EC_50_ values for single experiments (calculated by curve fitting) in scatter plots (*p* value from paired Student’s t-test).

**FIGURE 3:**
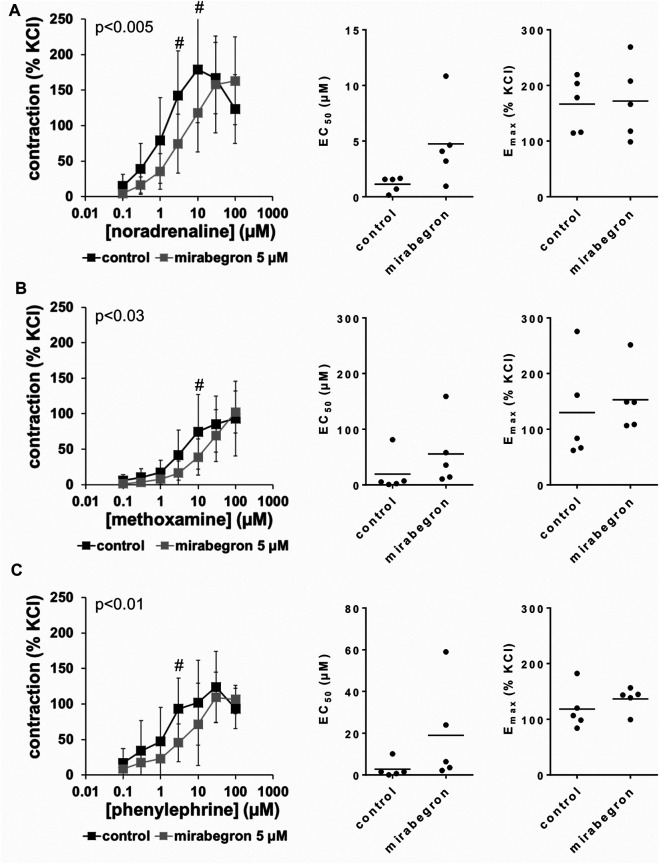
Effects of 5 µM mirabegron on α_1_-adrenergic contractions of human prostate tissues. Contractions of prostate tissues from the periurethral zone were induced by cumulative concentrations of noradrenaline **(A)**, methoxamine **(B)** or phenylephrine **(C)** in an organ bath, 30 min after addition of mirabegron or an equivalent amount of DMSO (controls), which was used as solvent for mirabegron. Shown are data from *n* = 5 patients in each diagram, which are means ± SD in frequency response curves (#*p* < 0.05 for control vs. mirabegron by two-way ANOVA, and *p* values for whole groups in inserts from 1-way ANOVA), and E_max_ and EC_50_ values for single experiments (calculated by curve fitting) in scatter plots.

**FIGURE 4:**
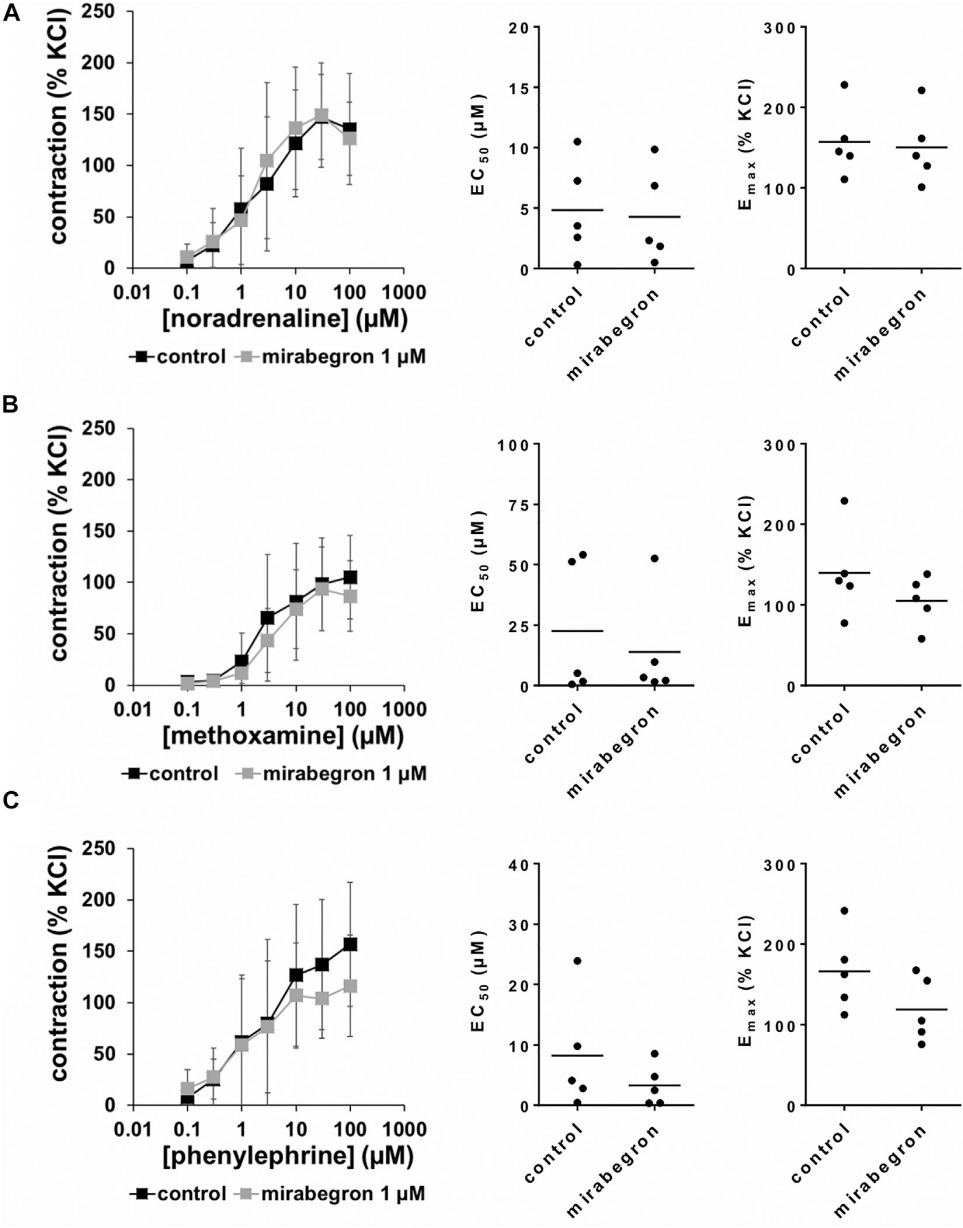
Effects of 1 µM mirabegron on α_1_-adrenergic contractions of human prostate tissues. Contractions of prostate tissues from the periurethral zone were induced by cumulative concentrations of noradrenaline **(A)**, methoxamine **(B)** or phenylephrine **(C)** in an organ bath, 30 min after addition of mirabegron or an equivalent amount of DMSO (controls), which was used as solvent for mirabegron. Shown are data from *n* = 5 patients in each diagram, which are means ± SD in frequency response curves, and E_max_ and EC_50_ values for single experiments (calculated by curve fitting) in scatter plots.

All three α_1_-adrenergic agonists (noradrenaline, methoxamine, phenylephrine) induced concentration-dependent contractions of human prostate tissues, resulting in sigmoidal concentration response curves in control groups. 10 and 5 μM, but not 1 µM of mirabegron caused rightshifts of concentration response curves of all three α_1_-adrenergic agonists (Figures 2–4). Rightshifts included inhibition of contractions at submaximum agonist concentrations, but recovery of contractions at high agonist concentrations (Figures 2, 3) (Table 2), and increased EC_50_ values, but unchanged E_max_ values calculated by curve fitting (Figures 2, 3).

**TABLE 2:** Mean differences (MD) for noradrenaline-, methoxamine- and phenylephrine-induced contractions of human prostate tissues after application of mirabegron (1, 5, 10 μM) or DMSO for controls, shown for each applied agonist concentration and with 95% confidence intervals (CI) (in square brackets, low to high) (% of KCl-induced contractions). Differences in contraction at given agonist concentrations were calculated for each single experiment (i. e., between mirabegron and control group, for corresponding, paired samples from the same prostate in each single experiment), and are expressed as MD with 95% CI.

Mirabegron (μM)	Noradrenaline concentration						
	0.1 µM	0.3 µM	1 µM	3 µM	10 µM	30 µM	100 µM
**1**	3.6 [−15.1 to 22.4]	3.5 [−48 to 55.1]	−10.9 [−107.4 to 85.5]	22.8 [−118 to 163.7]	14.6 [−104 to 133.3]	2.0 [−91.1 to 95]	−9.4 [−80.4 to 61.6]
**5**	−10.5 [−31.3 to 10.4]	−22.7 [−54.6 to 9.2]	−43.7 [−94.7 to 7.4]	−67.7 [−152.5 to -17.2]	−61.4 [−183.1 to 60.4]	−9 [−98 to 80]	39.6 [−3.6 to 82.8]
**10**	−17.6 [−35.3 to 0]	−61.6 [−108.9 to −14.3]	−69.2 [−132.2 to −6.3]	−76.6 [−135 to −18.7]	−53.5 [−107.1 to 0.2]	11 [−21.4 to 43.4]	14.3 [−14.9 to 43.4]
	**Methoxamine concentration**						
		**0.1 µM**	**0.3 µM**	**1 µM**	**3 µM**	**10 µM**	**30 µM**	**100 µM**
**1**	−1.6 [−4.9 to 1.8]	−0.5 [−4.9 to 4]	−11.4 [−39.7 to 16.9]	−22.3 [−70.8 to 26.2]	−7.4 [−52.8 to 38.1]	−4.7 [−33.9 to 24.6]	−18.5 [−40.4 to 3.4]
**5**	−4.4 [−12.7 to 3.9]	−6.9 [−19.3 to 5.5]	−9.1 [−20.3 to 2.1]	−25.3 [−50.7 to 0.02]	−35.9 [−69.7 to -2]	−16.3 [−48 to 15.4]	9.1 [−52 to 70.2]
**10**	−8.9 [−17.2 to -0.7]	−19.3 [−27.9 to −10.6]	−44.2 [−60.8 to −27.6]	−61.7 [−103 to −20.4]	−75.3 [−184.1 to 33.6]	−41.9 [−148.3 to 64.5]	−6.8 [−81.7 to 68.2]
	**Phenylephrine concentration**						
		**0.1 µM**	**0.3 µM**	**1 µM**	**3 µM**	**10 µM**	**30 µM**	**100 µM**
**1**	8.7 [−10.4 to 27.7]	2.2 [−20.9 to 25.2]	-2.8 [−60.5 to 54.8]	−3 [−72.3 to 66.3]	−19.9 [−64 to 24.3]	−32.9 [−86.3 to 20.6]	−40.6 [−76.2 to -5]
**5**	−8.4 [−22 to 5.3]	−16.7 [−44.7 to 11.4]	−24.2 [−55.1 to 6.7]	−47.9 [−74.8 to −21.1]	−30.5 [−90.2 to 29.3]	−14.7 [−57.9 to 28.5]	12.6 [−13.9 to 39.1]
**10**	−2.3 [−4.9 to 0.2]	−14.1 [−30.6 to 2.3]	−18.2 [−33.3 to −3.1]	−43.9 [−70.7 to −17.2]	−44 [−94.4 to 6.4]	−23.2 [−55.5 to 9.2]	0.6 [−57.2 to 58.3]

Data are based on experiments on series with *n* = 5 prostates for each mirabegron concentration and corresponding controls, i. e. 45 prostates in total.

Bold values are either concentrations of adrenergic agonists (horizontal, value with unit (=μM)), or concentrations of mirabegron (vertical, value, unit is above (=after“mirabegron”)).

After application of 10 µM mirabegron, contractions induced by noradrenaline, methoxamine, and phenylephrine at concentrations up to 10 µM were lower compared to corresponding control groups (Figure 2) (Table 2). At concentrations of α_1_-adrenergic agonists of 100 μM, contractions were similar after 10 µM mirabegron and in control groups (Figure 2) (Table 2). The EC_50_ for noradrenaline was increased by approximately one log unit, from 0.5 µM [−0.3 to 1.4] in controls to 5.1 µM [2.1 to 8.1] by 10 µM mirabegron (MD 4.5 µM [1.9 to 7.1]). The EC_50_ for phenylephrine was increased from 7.4 µM [−4.1 to 18.8] in controls to 29.1 µM [4.2 to 54.1] by 10 µM mirabegron (MD 21.9 µM [−1 to 44.6]). The EC_50_ for methoxamine was increased approximately 20-fold, from 3.9 µM [−3.9 to 11.7] in controls to 83.3 µM [−84.9 to 251.6] by 10 µM mirabegron (MD 79.4 µM [−60.4 to 219.3]).

After application of 5 µM mirabegron, contractions induced by noradrenaline, methoxamine, and phenylephrine at concentrations up to 10 µM were lower compared to corresponding control groups (Figure 3) (Table 2). At concentrations of α_1_-adrenergic agonists of 30–100 μM, contractions were similar after 5 µM mirabegron and in control groups (Figure 3) (Table 2). The EC_50_ for noradrenaline was increased from 1.1 µM [0.3 to two] in controls to 4.7 µM [0.2 to 9.3] by 5 µM mirabegron (MD 3.6 µM [−0.2 to 7.4]). The EC_50_ for phenylephrine was increased from 2.8 µM [−2.4 to 7.9] in controls to 19 µM [−10.8 to 48.8] by 5 µM mirabegron (MD 16.2 µM [−8.9 to 41.4]). The EC_50_ for methoxamine was increased from 19.4 µM [−23.6 to 62.4] in controls to 55.6 µM [−20 to 131.2] by 5 µM mirabegron (MD 36.2 µM [−36.1 to 108.4]).

After application of 1 µM mirabegron, no changes in concentration response curves were observed compared to corresponding controls, apart from slight decreases in contractions induced by 30 and 100 µM phenylephrine (Figure 4) (Table 2). In contrast to mirabegron at concentrations of 5 and 10 μM, mirabegron at a concentration of 1 µM did not increased EC_50_ values of α_1_-adrenergic agonists (Figure 4).

### Effects of Mirabegron on Non-adrenergic Contractions

**FIGURE 5:**
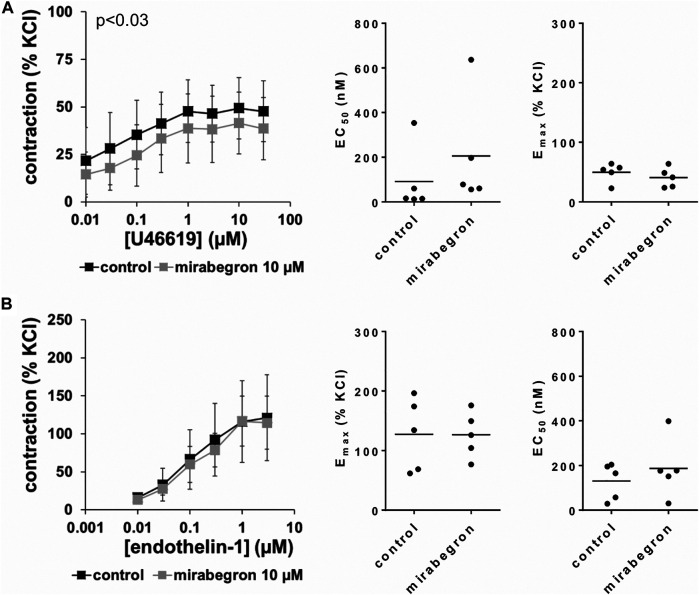
Effects of 10 µM mirabegron on non-adrenergic contractions of human prostate tissues. Contractions of prostate tissues from the periurethral zone were induced by cumulative concentrations of the thromboxane A_2_ analog U46619 **(A)** or endothelin-1 **(B)** in an organ bath, 30 min after addition of mirabegron or an equivalent amount of DMSO (controls), which was used as solvent for mirabegron. Shown are data from *n* = 5 patients in each diagram, which are means ± SD in frequency response curves (*p* value for whole groups in insert from 1-way ANOVA), and E_max_ and EC_50_ values for single experiments (calculated by curve fitting) in scatter plots.

U46619 and endothelin-1 induced concentration-dependent contractions of human prostate tissues (Figure 5). U46619-induced contractions were slightly inhibited by 10 µM mirabegron, reflected by concentration response curves and by increased EC_50_ values (Figure 5A) (Table 3). EC_50_ values for U46619 were approximately doubled, from 91 nM [−93 to 275] in controls to 206 nM [−102 to 513] by 10 µM mirabegron (MD 115 nM [−183 to 412]). However, E_max_ values calculated by curve fitting remained unchanged by 10 µM mirabegron (Figure 5A). Contractions induced by endothelin-1 remained unchanged by mirabegron, including concentrations response curves, EC_50_ values, and calculated E_max_ values (Figure 5B) (Table 3).

**TABLE 3:** Mean differences (MD) for U46619- and endothelin-1induced contractions after application of mirabegron (10 µM) or DMSO for controls, shown for each applied agonist concentration and with 95% confidence intervals (CI) (in square brackets, low to high) (% of KCl-induced contractions). Differences in contraction at given agonist concentrations were calculated for each single experiment (i. e., between mirabegron and control group, for corresponding, paired samples from the same prostate in each single experiment), and are expressed as MD with 95% CI.

	Concentration of contractile agonist							
	0.01 µM	0.03 µM	0.1 µM	0.3 µM	1 µM	3 µM	10 µM	30 µM
**U46619**	−7.1 [−37.6 to 23.3]	−10.2 [−41.4 to 21]	−11 [−44.3 to 22.4]	−7.9 [−40.6 to 24.8]	−9 [−41.1 to 23.1]	−8.3 [−32.4 to 15.8]	−7.8 [−29.9 to 14.2]	−9.2 [−30.1 to 11.8]
**Endothelin-1**	−3.1 [−15.1 to 8.8]	−5.6 [−37.3 to 26.1]	−6.5 [−39.1 to 26.1]	−13.8 [−49.3 to 21.8]	0.6 [−58.9 to 60.2]	−6.5 [−67.6 to 54.6]	—	—

Data are based on experiments on series with n = 5 prostates for each mirabegron concentration and corresponding controls, i. e. 10 prostates in total.

### Effects of Mirabegron on KCl-Precontracted Tissues

**FIGURE 6:**
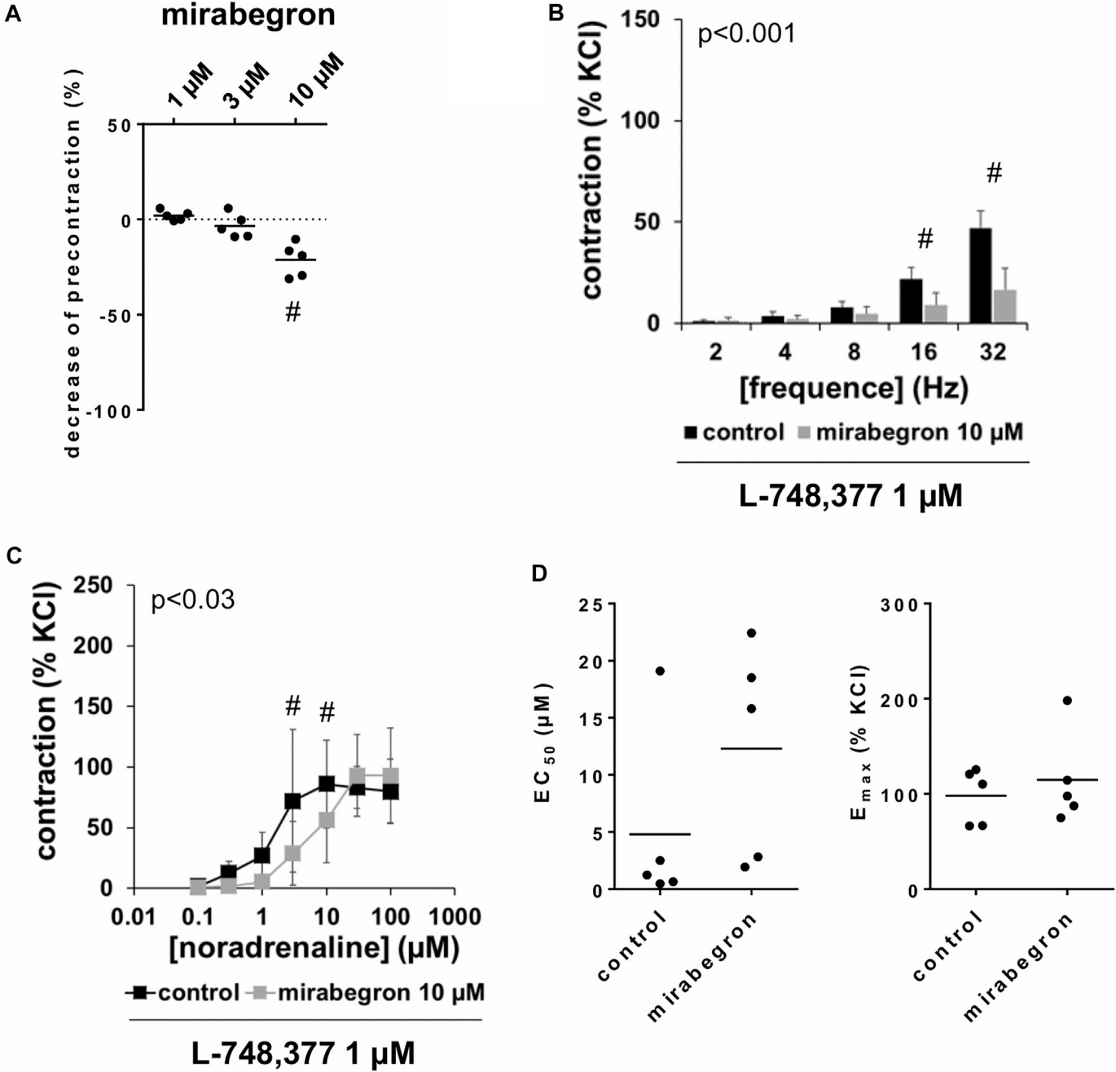
Effects of mirabegron on KCl-precontracted human prostate tissues, and on EFS- and noradrenaline-induced contractions in the presence of L-748,377. In **(A)**, prostate tissues were precontracted with 80 mM KCl. After a stable precontraction was attained, mirabegron was added in cumulative concentrations (1–10 µM). Shown are data from n = 5 patients together with means, which are expressed as percentage decrease from the KCl-induced precontraction (#*p* < 0.05 vs. precontraction before mirabegron by Dunn’s test). Note that KCl-induced contractions were set to 100% and all values before mirabegron reflect 0% relaxation (dotted line). In **(B)** and **(C)**, contractions of prostate tissues from the periurethral zone were induced by EFS **(B)** or noradrenaline **(C)** in an organ bath, 50 min after adddition of L-748,377 (1 µM) to all tissues and 30 min after addition of mirabegron (10 µM) or an equivalent amount of DMSO (controls), which was used as solvent for mirabegron. Shown are data from *n* = 5 patients in each diagram, which are means ± SD in frequency response curves (#*p* < 0.05 for control vs. mirabegron by two-way ANOVA, and *p* values for whole groups in inserts from 1-way ANOVA).

Following precontraction with 80 mM KCl and achievement of a stable precontraction, mirabegron was added in cumulative concentrations. At concentrations of 1 and 3 μM, both left for 5 min before the next concentration was added, mirabegron did not change KCl-induced tensions (Figure 6A). Thus, average tensions amounted to 102% of KCl precontraction [99 to 105] after 1 µM mirabegron, and to 97% of KCl precontraction [89 to 104] after 3 µM mirabegron. After addition of 10 µM mirabegron, moderate decreases of KCl precontraction were observed (Figure 6A), appoaching to an average tension of 79% of KCl precontraction [68 to 90] within 10 min, i. e. to the end of the experiment.

### Effects of L-748,377 on Mirabegron-Induced Contraction Inhibition

Frequence-dependent contractions by EFS and concentration-dependent contractions by noradrenaline were induced following application of 10 µM mirabegron or a corresponding amount of DMSO, but all in the presence of L-748,377 (1 µM). Similar to experiments without L-748,377 (3.1, 3.2), mirabegron inhibited EFS-induced contractions, and caused rightshifts of concentration response curves for noradrenaline. Again, inhibitions of EFS-induced contractions were clearest at frequencies of 16 and 32 Hz (Table 4). In the presence of L-748,377, contractions were inhibited by 60% [34 to 85] at 16 Hz and by 65% [41 to 90] at 32 Hz by 10 µM mirabegron (Figure 6B). Rightshifts of concentration response curves for noradrenaline included inhibition of contractions at noradrenaline concentration up to 10 μM, but recovery of contractions at high agonist concentrations (Figure 6C) (Table 5), and increased EC_50_ values, but unchanged E_max_ values calculated by curve fitting (Figure 6C) in the presence of L-748,377. At noradrenaline concentrations of 30 and 100 μM, contractions were similar after 10 µM mirabegron and in control groups (Figure 6C) (Table 5). The EC_50_ for noradrenaline was increased from 4.8 µM [−5.2 to 14.8] in controls to 12.3 µM [0.7 to 23.9] by 10 µM mirabegron in the presence of L-748,377 (MD 7.5 µM [−5.2 to 20.2]).

**TABLE 4:** Mean differences (MD) for EFS-induced contractions of human prostate tissues after application of either L-748,337, l-NAME, or BPIPP both to control and mirabegron groups, followed by application of mirabegron (10 µM) or DMSO for controls, shown for each applied frequency and with 95% confidence intervals (CI) (in square brackets, low to high) (% of KCl-induced contractions). Differences in contraction at given frequencies were calculated for each single experiment (i. e., between mirabegron and control group, for corresponding, paired samples from the same prostate in each single experiment), and are expressed as MD with 95% CI.

	EFS, frequence				
	2 Hz	4 Hz	8 Hz	16 Hz	32 Hz
**L-748,337**	0.4 [−1.5 to 2.2]	−1.6 [−4.6 to 1.3]	−3.3 [−8.2 to 1.6]	−12.6 [−21.4 to −3.9]	−30.1 [−44.3 to −15.9]
**l** **-NAME**	−3 [−8.9 to 2.9]	−4.4 [−12.9 to 4.1]	−6.1 [−16.6 to 4.4]	−10.9 [−25.4 to 3.6]	−36.1 [−58.7 to −13.4]
**BPIPP**	−1.8 [−4.6 to 1]	−3.5 [−6.9 to -0.2]	−8.7 [−15.6 to -1.8]	−18.2 [−25.6 to −10.8]	−32.2 [−41.7 to −21.6]

Data are based on experiments on series with n = 5 prostates for each setting, i. e. 15 prostates in total.

**TABLE 5:** Mean differences (MD) for noradrenaline-induced contractions of human prostate tissues after application of either L-748,337, l-NAME, or BPIPP both to control and mirabegron groups, followed by application of mirabegron (10 µM) or DMSO for controls, shown for each applied agonist concentration and with 95% confidence intervals (CI) (in square brackets, low to high) (% of KCl-induced contractions). Differences in contraction at given agonist concentrations were calculated for each single experiment (i. e., between mirabegron and control group, for corresponding, paired samples from the same prostate in each single experiment), and are expressed as MD with 95% CI. Data are based on experiments on series with *n* = 5 prostates for each mirabegron concentration and corresponding controls with L-748,337 and l-NAME and *n* = 9 prostates with BPIPP, i. e. 19 prostates in total.

	Noradrenaline concentration						
	0.1 µM	0.3 µM	1 µM	3 µM	10 µM	30 µM	100 µM
**L-748,337**	−1 [−2.1 to 0]	−10.7 [−21.1 to −0.2]	−21.4 [−41.8 to −1]	−43.5 [−109.7 to 22.8]	−29.9 [−81.9 to 22.1]	9.8 [−29.4 to 48.9]	13.2 [−35.7 to 62.1]
**l** **-NAME**	−5.5 [−11.9 to 0.8]	−18.6 [−30 to −7.3]	−32.3 [−52.4 to −12.1]	−78.4 [−136 to −20.9]	−74.5 [−130 to −18.6]	−20 [−88.8 to 48.8]	−11.6 [−80.9 to 57.7]
**BPIPP**	−5.5 [−11.6 to 0.7]	−16.6 [−34.9 to 1.7]	−21.1 [−42.2 to 0]	−20.8 [−52.4 to 10.7]	−22.2 [−57 to 12.6]	−11.4 [−47.8 to 24.9]	−3.7 [−33.4 to 26]

### Effects of L-NAME and BPIPP on Mirabegron-Induced Contraction Inhibition

**FIGURE 7:**
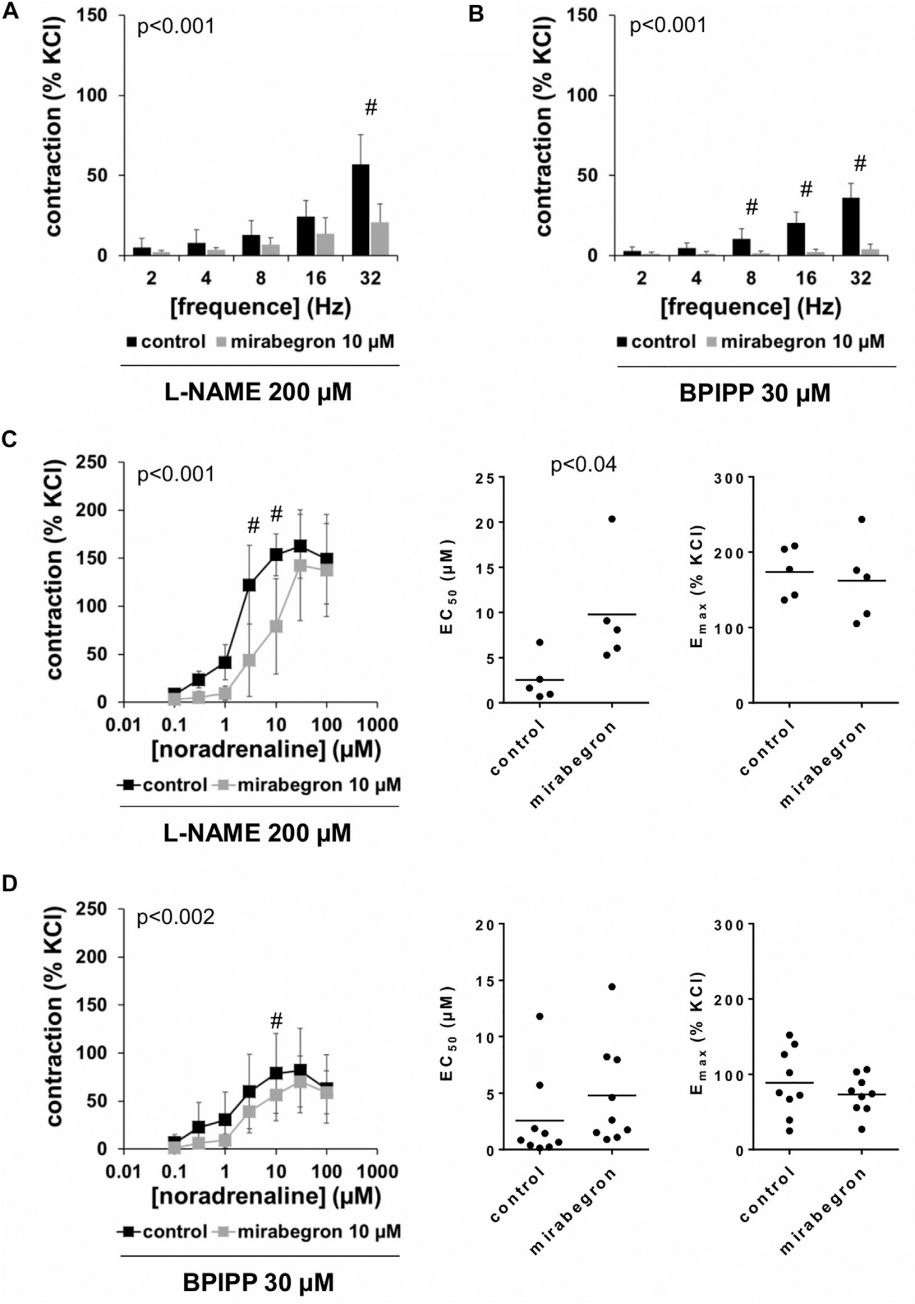
Effects of mirabegron on EFS- and noradrenaline-induced contractions of human prostate tissues in the presence of l-NAME or BPIPP. Contractions of prostate tissues from the periurethral zone were induced by EFS **(A, B)** or noradrenaline **(C, D)** in an organ bath, 50 min after adddition of either l-NAME (200 µM) **(A, C)** or BPIPP (30 µM) **(B, D)** to all tissues and 30 min after addition of mirabegron (10 µM) or an equivalent amount of DMSO (controls), which was used as solvent for mirabegron. Shown are data from *n* = 5 patients in each diagram in **(A–C)** and from *n* = 9 patients in **(D)**, which are means ± SD in frequency response curves (#*p* < 0.05 for control vs. mirabegron by two-way ANOVA, and *p* values for whole groups in inserts from 1-way ANOVA).

Frequence-dependent contractions by EFS and concentration-dependent contractions by noradrenaline were induced following application of 10 µM mirabegron or a corresponding amount of DMSO, but all in the presence of either L-NAME (200 µM) or BPIPP (30 µM) in different series. Similar to experiments without L-NAME or BPIPP (3.1, 3.2), mirabegron inhibited EFS-induced contractions in the presence of L-NAME or BPIPP. Again, inhibitions of EFS-induced contractions were clearest at frequencies of 16 and 32 Hz (Table 4). In the presence of L-NAME, EFS-induced contractions were inhibited by 47% [22 to 72] at 16 Hz and by 59% [24 to 95] at 32 Hz by 10 µM mirabegron (Figure 7A). In the presence of BPIPP, EFS-induced contractions were inhibited by 89% [80 to 98] at 16 Hz and by 89% [82 to 96] at 32 Hz by 10 µM mirabegron (Figure 7B). Rightshifts of concentration response curves for noradrenaline by mirabegron were obvious in the presence of L-NAME, and still persisted, although less proncounced, in the presence of BPIPP (Figures 7C,D). Using both compounds, rightshifts included inhibition of contractions at noradrenaline concentration up to 10 μM, but recovery of contractions at high agonist concentrations (Figures 7C,D) (Table 5), and increased EC_50_ values, but unchanged E_max_ values calculated by curve fitting (Figures 7C,D). At noradrenaline concentrations of 30 and 100 μM, contractions in the presence of either L-NAME or BPIPP were similar after 10 µM mirabegron and in control groups (Figures 7C,D) (Table 4). In the presence of L-NAME, the EC_50_ for noradrenaline was increased from 2.5 µM [−0.5 to 5.6] in controls to 9.8 µM [2.2 to 17.4] by 10 µM mirabegron (MD 7.3 µM [0.5 to 14]). In the presence of BPIPP, the EC_50_ for noradrenaline was increased from 2.6 µM [−0.4 to 5.5] in controls to 4.8 µM [1.3 to 8.3] by 10 µM mirabegron (MD 2.2 µM [−6.5 to 2]). The E_max_ for noradrenaline-induced contractions was reduced from 89% of KCl-induced contractions [55 to 123] in controls to 73% of KCl-induced contractions [54 to 93] by 10 µM mirabegron in the presence of BPIPP (MD 15 percentage points [−52 to 20]).

### Effects of Miragebron on Viability, Proliferation and cAMP Formation in Stromal Cells

**FIGURE 8:**
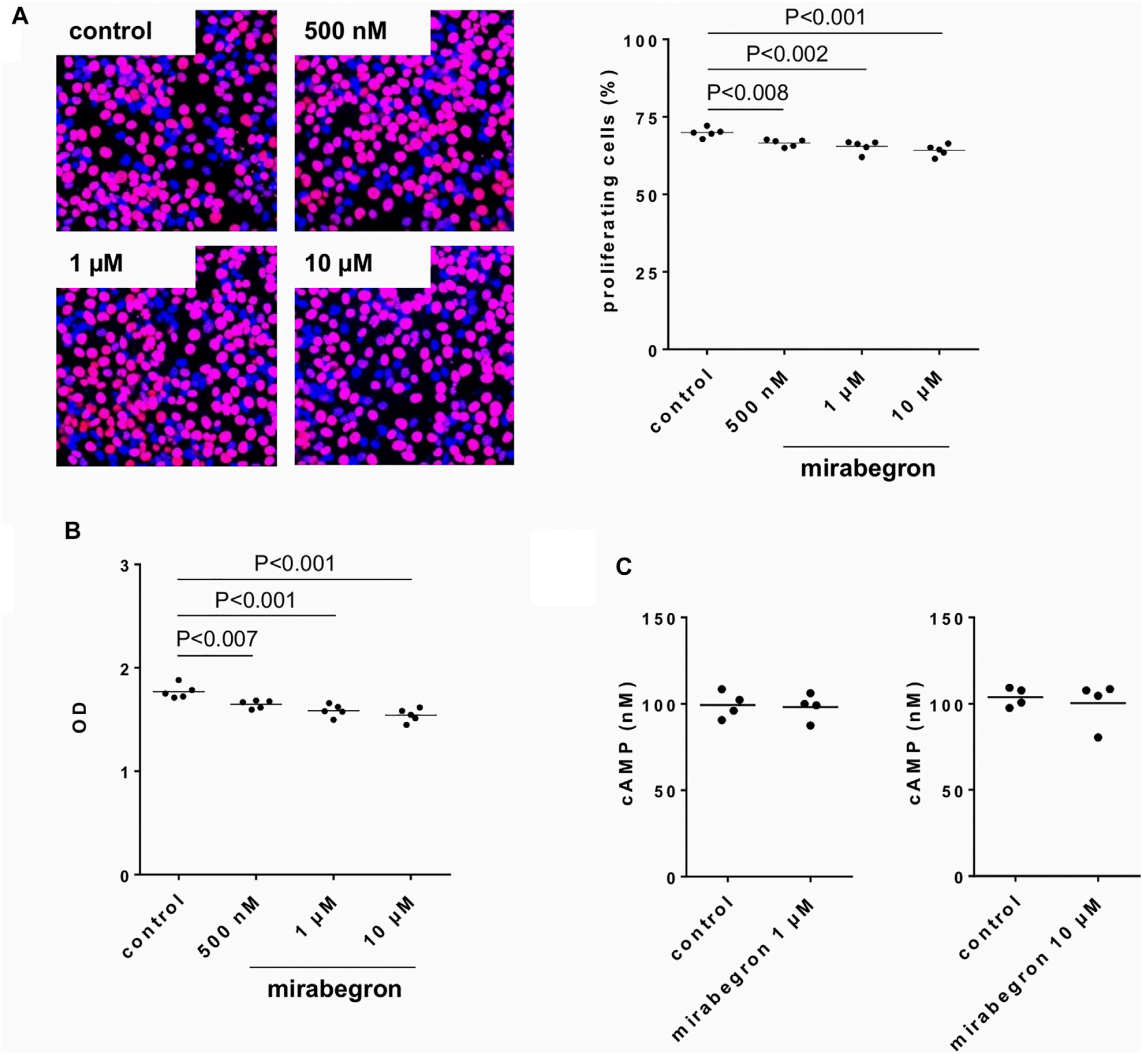
Effects of mirabegron on proliferation, viability and cAMP formation in human prostate stromal cells. In **(A)**, proliferation was assessed by EdU assays (red, proliferating cells; blue, non-proliferating cells), following incubation of WPMY-1 cells with DMSO (controls) or mirabegron in indicated concentrations for 24 h. Proliferation is expressed as percentage of cells showing proliferation, referred to the number of all cells in a microscopic field. In **(B)**, viability was assessed by CCK-8 assays, following incubation of WPMY-1 cells with DMSO (controls) or mirabegron in indicated concentrations for 48 h. Viability is expressed as extinction (OD), which is the final readout of CCK-8 assays. In **(C)**, cAMP in lysates was assessed by ELISA following incubation of cultured stromal cells with 1 µM or 10 µM mirabegron for 30 min, and in corresponding controls. In **(A)** and **(B)**, all single values together with means and *p* values (Dunnett’s test) from five independent experiments for each setting are shown, together with pictures from represenative experiments from EdU assay. In **(C)**, all single values with means from four independent experiments are shown.

Proliferation remained virtually unaffected by application of mirabegron in concentrations of 500 nM, 1 and 10 µM for 24 h, although slight decreases in average proliferation indexes occured (Figure 8A). Compared to controls, proliferation rate was decreased by 4.8% [2.5 to 7] by 500 nM mirabegron, 6.4% [1.3 to 11.5] by 1 µM mirabegron, and 8.2% [3.5 to 12.8] by 10 µM mirabegron. Similarly, the viability remained virtually unaffected by application of mirabegron in concentrations of 500 nM, 1 and 10 µM for 48 h, although slight decreases occured (Figure 8B). Compared to controls, viability was decreased by 11.5% [6.9 to 16.1] by 500 nM mirabegron, 14.8% [8.7 to 20.8] by 1 µM mirabegron, and 17.2% [11.4 to 22.9] by 10 µM mirabegron. Neither 1 µM mirabegron, nor 10 µM mirabegron, both applied for 30 min, changed the cAMP levels in cultured stromal cells (Figure 8C). cAMP concentrations in lysates amounted to 98 nM [86 to 111] after incubation with 1 µM mirabegron and to 99 nM [87 to 112] in corresponding controls, and to 100 nM [79 to 122] after incubation with 10 µM mirabegron and to 104 nM [95 to 113] in corresponding controls.

## Discussion

The findings of this study suggest that mirabegron may act as an α_1_-adrenoceptor antagonist on adrenergic smooth muscle contractions and inhibits neurogenic contractions in the human prostate, using concentrations of 5 µM or higher *in vitro*. In contrast, mirabegron did not show effects using 1 μM, or on non-adrenergic contractions or proliferation of stromal cells. Obviously, effects of mirabegron in the human prostate are limited to off-target effects and require high concentrations, while β_3_-adrenoceptor-specific effects are obviously lacking.

In our contraction experiments using α_1_-adrenergic agonists, mirabegron showed typical characteristics of an antagonist, including rightshifts of concentration response curves, increased EC_50_ values for α_1_-adrenergic agonists, and unchanged E_max_ values. These features were observed and confirmed using three different α_1_-adrenergic agonists. Curve fitting was not possible for EFS-induced contractions, due to lacking sigmoidal character of frequence repsonse curves. EFS-induced contractions of human prostate tissues are supposed to mediated by release of endogenous neurotransmitters and subsequent α_1_-adrenergic contractions (Yu et al., 1994; Angulo et al., 2012; Spek et al., 2021). Thus, the inhibition of EFS-induced contractions by miragebron may result from antagonism of α_1_-adrenoceptors as well. As effects on endothelin-1- or U46619-induced contractions were lacking or neglectable despite reaching significance level, effects of mirabegron in our experiments were obviously largely or completely limited to antagonism of α_1_-adrenoceptors. We were particular interested in addressing the effects on these non-adrenergic inhibitors, because they induce contractions of prostate smooth muscle in parallel to α_1_-adrenoceptors and have been suspected to account for limitations of α_1_-blockers, which are well-known from LUTS treatment (Hennenberg et al., 2014; Hennenberg et al., 2017; Yu et al., 2019). Our findings are in line with observations from mouse urethra smooth muscle, where mirabegron did not inhibit endothelin-1- or vasopressin-induced contractions (Alexandre et al., 2016).

To confirm that the observed inhibition of EFS-induced contractions and the rightshifts of noradrenaline-induced contractions by mirabegron were not imparted by activation of β_3_-adrenoceptors, we repeated these experiments in the presence of L-748,377 in all samples, i. e. in samples with mirabegron and DMSO. The effect of mirabegron turned out as resistant to L-748,377, supporting the idea that the inhibition and rightshifts of contractions are rather caused by antagonism of α_1_-adrenoceptors than by activation of β_3_-adrenoceptors. We applied L-748,377 in a concentration of 1 μM, which should antagonize β_3_-and β_2_-, and (at least partially) β_1_-adrenoceptors, considering reported K_i_ values of 4, 204 and 390 nM for β_3_-, β_2_-and β_1_-adrenoceptors (Candelore et al., 1999). An involvement of β_1_-adrenoceptors appears unlikely anyway, as the presumed relevance of this subtype is low or neglectable in prostate smooth muscle (Michel and Vrydag, 2006). β-Adrenoceptors, including β_3_-adrenoceptors may act via cAMP, or via nitric oxide-induced cGMP formation, which may both cause smooth muscle relaxation (Flacco et al., 2013; Bussey et al., 2018). Accordingly, we assessed impacts of the NOS inhibitor l-NAME, and an inhibitor of guanylyl and adenylyl cyclases, BPIPP on mirabegron effects. Again, inhibition of EFS-induced contractions were obvious in the presence of l-NAME or BPIPP. Similarly, rightshifts of concentration response curves were observed in the presence of both compounds, although being clearest with l-NAME and less pronounced under BPIPP. Consequently an involvement of β-adrenoceptors in mirabegron effects appears unlikely. In line with these findings from functional experiments using prostate tissues, mirabegron did not increase cAMP levels in cultured stromal cells, using concentrations of 1 µM or 10 µM applied for the same period as in the organ bath, i. e. 30 min. Finally, mirabegron did not induce relaxation of KCl-precontracted tissues at concentrations of 1 µM or 3 μM, which should be expected if it acts by β_3_-adrenoceptor-induced cAMP formation. Whether the small relaxant effects observed after 10 µM mirabegron and 20 min after application of the first concentration represent a mirabegron effect (regardless whether β_3_-adrenoceptor-specific or by other mechanisms) or reflects spontaneous decreases of tension, can be hardly estimated, as no controls of KCl-induced contractions but without mirabegron were included.

We applied different concentrations of mirabegron, and observed no effects using 1 μM, but obvious effects using 5 μM, and strong effects using 10 µM mirabegron. Together, this points to an EC_50_ and K_D_ value of mirabegron binding to α_1_-adrenoceptors, which is far higher than previously reported β_3_-adrenoceptor-specific EC_50_ and K_i_ values. Mirabegron has been described and introduced as a β_3_-adrenoceptor agonist. Binding assays pointed to K_i_ values of 2.5 nM for human β_3_-adrenoceptors, and 383 and 977 nM for human β_1_-and β_2_-adrenoceptors, respectively (Tasler et al., 2012). In chinese hamster ovary (CHO) cells transfected with human β_3_-adrenoceptors, mirabegron induced cAMP formation with an EC_50_ of 22.4 nM, while EC_50_ values ranged higher than 10 µM following transfection with β_1_-or β_2_-adrenoceptors (Takasu et al., 2007). Similar values by the same assays were reported for rat β-adrenoceptors, amounting to EC_50_ values for mirabegron of 19 nM with rat β_3_-adrenoceptors, 610 nM with β_1_-adrenoceptors, and undeterminable, because sumless EC_50_ with β_2_-adrenoceptors (Hatanaka et al., 2013). *In vitro*, mirabegron relaxed human and non-human detrusor tissues, with EC_50_ values depending on precontraction, species, and disease. Following precontraction with 1 µM carbachol, mirabegron-induced relaxations were observed with EC_50_ values of 588 nM in normal detrusor tissues, 912 nM in detrusor tissues from patients with bladder outlet obstruction (BOO), but 3.89 µM in detrusor tissues from patients with BOO and detrusor overactivity (DO), with maximum relaxations of 28–36% (Svalo et al., 2013). Another study reported EC_50_ values of 780 nM for relaxation of human bladder strips precontracted with 100 nM carbachol, and of 5.1 µM for relaxation of rat bladder strips precontracted with 1 µM carbachol (Takasu et al., 2007). Together, these observations suggested β_3_-adrenoceptor-mediated relaxations in the bladder, and were the basis for clinical trials addressing the effects of mirabegron on storage symptoms in patients with OAB and subsequent approval for this indication.

Our findings are rather in line with previous studies reporting effects and α_1_-adrenergic antagonism of mirabegron in several smooth muscle-rich organs, than with studies reporting β_3_-adrenergic relaxations by mirabegron. Thus, concentration response curves for phenylephrine-induced contractions of mouse urethral tissues were shifted right concentration-dependently by mirabegron, with 0.1 µM mirabegron showing no effects, 1 µM mirabegron showing moderate shifts, and 30 µM increasing the EC_50_ of phenylephrine by approximately 1.5 magnitudes (Alexandre et al., 2016). Consequently, higher concentrations were used for other tissues in the same study, resulting in clear shifts by the lowest applied mirabegron concentration of 10 μM, and again increases in EC_50_ values of approximately 1.5 magnitudes for noradrenaline-induced contractions of rat vas deferens and rat aorta, and for phenylephrine-induced contractions of rat prostate tissues by 100 µM mirabegron (Alexandre et al., 2016). Similar to our data, concentration response curves fully recovered at higher concentrations of α_1_-adrenergic agonists, and no decreases in maximum contractions were observed (Alexandre et al., 2016). K_i_ values of mirabegron are available for all three subtypes of α_1_-adrenoceptors from competition assays using membranes of HEK-293 cells, and point to binding constants of 0.437 µM for α_1A_-, 1.8 µM for α_1D_-, and 26 µM for α_1B_-adrenoceptors (Alexandre et al., 2016). This affinity pattern is in line with the functional data, taking the organ-specific subtype expression into account, imparting α_1A_-mediated contractions in the urethra, prostate and vas deferens, and α_1D_-mediated contractions in the aorta (Alexandre et al., 2016). In the human and non-human prostate, α_1A_ is the predominant subtype, and accounts mostly, if not exclusively for α_1_-adrenergic contractions (Michel and Vrydag, 2006; Hennenberg et al., 2014). According to the low affinity of mirabegron for α_1B_-adrenoceptors in binding assays, α_1B_-mediated contractions in the rat spleen were not affected even by 100 µM mirabegron (Alexandre et al., 2016).

Previous findings from human prostate tissues are, to the best of our knowledge, limited to one series of experiments in a study addressing effects of mirabegron in rabbit and human prostates. Therein, 1 and 10 µM caused obvious inhibitions of maximum contractions of human prostate tissues induced by phenylephrine (Calmasini et al., 2015). In parallel, and similar to our findings, 10 µM increased the EC_50_ of phenylephrine from 5.7 to 35 μM, while 1 µM mirabegron was without effect on EC_50_ (Calmasini et al., 2015). EFS-induced contractions of rabbit prostate tissues were inhibited by 10 µM mirabegron, to similar extent as in our study using human tissues, but not inhibited by 1 µM mirabegron (Calmasini et al., 2015). However, and in contrast to our and other previous data suggesting no effects from lower mirabegron concentrations in the prostate, relaxation of phenylephrine-precontracted rabbit prostate tissues by mirabegron have been reported and pointed to an EC_50_ of mirabegron around 1 µM (Calmasini et al., 2015). Tissues in our study were sampled from the transitional, periurethral zone, which has been supposed to contribute most of all three prostate zones to urethral obstruction and voiding symptoms (Pradidarcheep et al., 2011). Growth of the transitional zone underlies BPH, but also occurs throughout life (Pradidarcheep et al., 2011; Timms and Hofkamp, 2011). However, patterns of contractility and smooth muscle tone differ between the three prostate zones and change with age (Lee et al., 2017; Chakrabarty et al., 2019). Consequently, our findings can not be necessarily extrapolated to the central or peripheral zones and do not cover all age groups, what may represent putative limitations of our study.

Although compounds antagonizing α_1_-adrenoceptors may be generally attractive in the context of BPH and are often considered as promising candidates for drug treatment of voiding symptoms, this does certainly not apply here. Taking our current and previous findings using human prostate tissues *in vitro* into account, any beneficial effect of mirabegron on voiding symptoms would require concentrations of 5 µM or higher, but could not be expected using 1 µM. However, maximum plasma concentrations of mirabegron after therapeutic dosing (50 mg daily) do not exceed 137 nM in men (Krauwinkel et al., 2012; Dehvari et al., 2018; Igawa et al., 2019). Accordingly, effects of mirabegron on voiding or voiding symptoms were reported only rarely, and most studies suggested no effects on urinary flow rate or prostate symptom scores (Nitti et al., 2013; Liao and Kuo, 2018). On the other hand, improvements of urinary flow rate and total symptom scores have been reported following add-on of mirabegron (50 mg/d) to α_1_-blockers in patients with mixed LUTS and α_1_-blocker-resistant symptoms, reaching similar values known for α_1_-blockers alone (Matsuo et al., 2016; Kang and Chung, 2020). Both studies were not placebo-controlled and did not include more than 50 patients. Consequently, these findings were not confirmed by a placebo-controlled, double-blinded phase four study, suggesting that add-on of mirabegron to an α_1_-blocker does not result in clinical meaningful improvements of maximum urinary flow rate (Herschorn et al., 2021). Plasma levels corresponding to a concentration of 5 µM *in vitro* and calculable effects on voiding symptoms would require dosages highly exceeding the established standard dosages of 50–100 mg daily. Safety and plasma levels of mirabegron have been addressed in phase I studies using daily doses up to 300 mg, which was well tolerated and resulted in maximum plasma concentrations of 961 nM in men (Krauwinkel et al., 2012). Extrapolating to a plasma concentration of 5 µM (which is the concentration, where antagonism was observed in our *in vitro* experiments), daily doses around 1,500 mg would be required to obtain such plasma levels, which have not yet been examined in safety studies. Thus, a physiological or clinical relevance of α_1_-adrenoceptor antagonism by mirabegron appears unlikely.

Thus, α_1_-adrenoceptor antagonism by mirabegron in the human prostate requires concentrations ranging around two log units higher than the K_i_ values in biochemical assays or than plasma concentrations during therapeutic dosing. Based on recent extrapolations, discrepancies of such extent are not sufficient to translate to clinically noticable effects. Thus, it has been proposed that β-adrenergic ligands showing binding to α_1_-adrenoceptors will only show α_1_-adrenoceptor-dependent effects, if the affinity difference is below one log unit, and higher differences will result in a receptor occupation of less than 10% (Michel, 2020). In fact, similar dual actions have been observed for several β-adrenoceptor ligands, in addition to mirabegron, so that such estimations are in fact possible (Michel, 2020).

It has been repeatedly proposed that adrenoceptors may promote and regulate proliferation and growth of different cell types. Findings from cell culture studies, animal models, and analyses of human tissues from patients being treated with α_1_-blockers suggested that α_1_-adrenoceptors induce proliferation and suppress apoptosis (Golomb et al., 1998; Kyprianou et al., 1998; Chon et al., 1999; Glassman et al., 2001; Turkeri et al., 2001; Anglin et al., 2002; Erdogru et al., 2002; Marinese et al., 2003; Kojima et al., 2009; Chagas-Silva et al., 2014; Nascimento-Viana et al., 2019; Liu et al., 2020). Proliferation and growth induced by β_3_-adrenoceptors has been proposed for several cell types, including few studies addressing smooth muscle and vascular cells (Hadi et al., 2013; Garcia-Alvarez et al., 2016). Accordingly, effects of mirabegron on proliferation and viability of prostate stromal cells appeared theoretically possible to us, so that we carried out experiments addressing the effects of mirabegron in WPMY-1 cells. Although some slight decays in proliferation and viability occured and were even significant, their extent was rather neglectable and too small to assume any clinically relevance. Thus, on the basis of our data, any effect of mirabegron on stromal growth or prostate size can not be expected *in vivo*, even not at dosages resulting in antagonism of α_1_-adrenoceptors (i. e. 50–100-fold of clinically used dosage). In fact, the clinical relevance of α_1_-adrenergic prostate growth, seen in experimental models is obviously limited. Based on a high number of clinical trials, there is a broad consensus that α_1_-blockers do not reduce prostate size in patients with BPH (Oelke et al., 2013). However, we did not examine effects on growth of glandular epithelial cells, which may take part in hyperplastic growth as well (Strand et al., 2017), so that any effect of mirabegron on prostate growth can not be fully excluded on the basis of our findings.

## Conclusion

Mirabegron inhibits neurogenic and α_1_-adrenergic human prostate smooth muscle contractions. This inhibition may be based on antagonism of α_1_-adrenoceptors by mirabegron, and does probably not include activation of β_3_-adrenoceptors and requires concentrations ranging 50–100 fold higher than plasma concentrations reported from normal dosing. Non-adrenergic contractions and proliferation of prostate stromal cells are not inhibited by mirabegron.

## Data Availability

The original contributions presented in the study are included in the article/Supplementary Material, further inquiries can be directed to the corresponding author.
